# Supplementary material for: Comprehensive circular RNA expression profile in radiation-treated HeLa cells and analysis of radioresistance-related circRNAs
Source: PeerJ. 2018 Jun 15;6:e5011. doi: 10.7717/peerj.5011 (PMC6005163; doi:10.7717/peerj.5011)
Supplement: Supplemental Information 3 [file peerj-06-5011-s003.docx]

Hela Cell STR Authentication Report

**MATERIALS AND METHODS**

PureLink® Genomic DNA Mini Kit (U.S. Life K182001) was used to the proper amount of Hela cell(1×10^6^) to extract genomic DNA (gDNA). It was amplified by using the PowerPlex®18D system (U.S. Promega DC1802) kit and tested by ABI3500 Genetic Analyzer (U.S. Life 3500).

**RESULTS**

Negative and positive results were correct.

The HeLa cell genotyping results of STR locus and Amelogenin locus are shown in the attached tables and the genetic map are shown in attached figures.

The genotyping results is satisfactory and genetic map is in high definition.

**Conclusion**

1. The STR genotyping result of Hela cell shows no human cell cross-contamination.
2. The STR genotyping of this cell strain can 100% matched with “Hela cell” in ATCC.

Table 1 The HeLa cell genotyping results of STR locus and Amelogenin locus

| HeLa cell | | |
| --- | --- | --- |
| Marker | Allele 1 | Allele 2 |
| D3S1358 | 15 | 18 |
| **THO1** | 7 | 7 |
| D21S11 | 27 | 28 |
| D18S51 | 16 | 16 |
| Penta E | 7 | 17 |
| **D5S818** | 11 | 12 |
| **D13S317** | 12 | 13.3 |
| **D7S820** | 8 | 12 |
| **D16S539** | 9 | 10 |
| **CSF1PO** | 9 | 10 |
| Penta D | 8 | 15 |
| **AMEL** | X | X |
| **Vwa** | 16 | 18 |
| D8S1179 | 12 | 13 |
| **TPOX** | 8 | 12 |
| FGA | 18 | 21 |
| D19S433 | 13 | 14 |
| D2S1338 | 17 | 17 |
| D12s391 | 20 | 25 |
| D6S1043 | 18 | 18 |


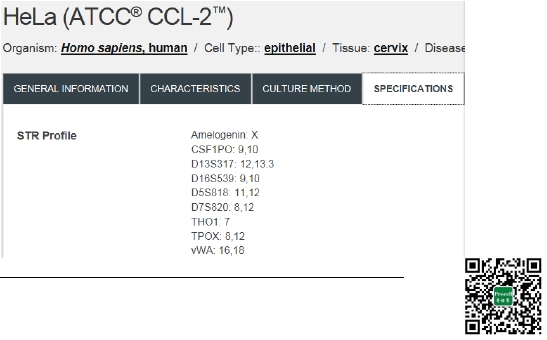


Figure1 The STR genotyping of “Hela cell” in ATCC.


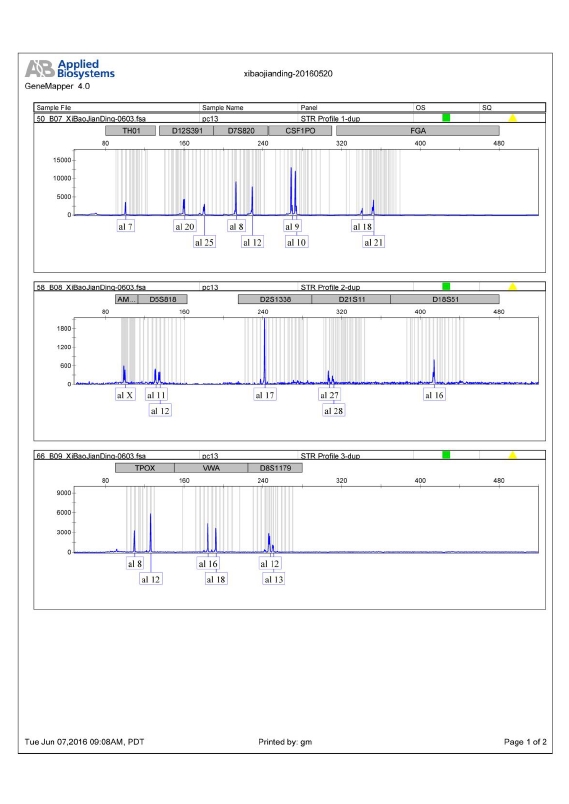

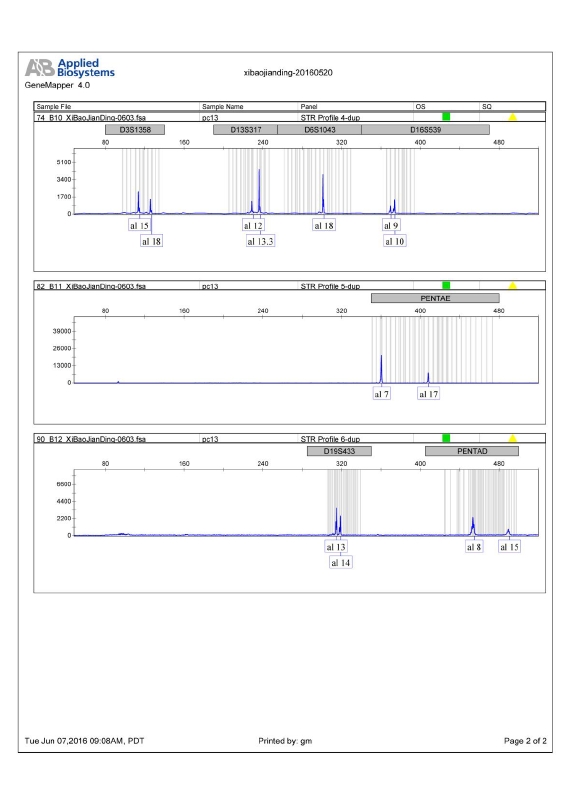


Figure2 The HeLa cell genetic map of STR locus and Amelogenin locus
